# Inducing Cold-Sensitivity in the Frigophilic Fly *Drosophila montana* by RNAi

**DOI:** 10.1371/journal.pone.0165724

**Published:** 2016-11-10

**Authors:** Felipe M. Vigoder, Darren J. Parker, Nicola Cook, Océane Tournière, Tanya Sneddon, Michael G. Ritchie

**Affiliations:** 1 Centre for Biological Diversity, School of Biology, University of St Andrews, Fife, United Kingdom; 2 Departamento de Genética, Universidade Federal do Rio de Janeiro, Rio de Janeiro, Brazil; 3 Department of Biological and Environmental Science, University of Jyväskylä, Jyväskylä, Finland; 4 Sars International Centre for Marine Molecular Biology, Thormøhlensgt, Bergen, Norway; Oxford Brookes University, UNITED KINGDOM

## Abstract

Cold acclimation is a critical physiological adaptation for coping with seasonal cold. By increasing their cold tolerance individuals can remain active for longer at the onset of winter and can recover more quickly from a cold shock. In insects, despite many physiological studies, little is known about the genetic basis of cold acclimation. Recently, transcriptomic analyses in *Drosophila virilis* and *D*. *montana* revealed candidate genes for cold acclimation by identifying genes upregulated during exposure to cold. Here, we test the role of *myo-inositol-1-phosphate synthase (Inos)*, in cold tolerance in *D*. *montana* using an RNAi approach. *D*. *montana* has a circumpolar distribution and overwinters as an adult in northern latitudes with extreme cold. We assessed cold tolerance of dsRNA knock-down flies using two metrics: chill-coma recovery time (CCRT) and mortality rate after cold acclimation. Injection of dsRNA*Inos* did not alter CCRT, either overall or in interaction with the cold treatment, however it did induced cold-specific mortality, with high levels of mortality observed in injected flies acclimated at 5°C but not at 19°C. Overall, injection with dsRNA*Inos* induced a temperature-sensitive mortality rate of over 60% in this normally cold-tolerant species. qPCR analysis confirmed that dsRNA injection successfully reduced gene expression of *Inos*. Thus, our results demonstrate the involvement of *Inos* in increasing cold tolerance in *D*. *montana*. The potential mechanisms involved by which *Inos* increases cold tolerance are also discussed.

## Introduction

Most ectothermic organisms adjust their physiology in response to gradual changes in environmental temperature. Such physiological changes can increase their tolerance to extreme seasonal temperatures allowing them to maintain function under predictable conditions [1–3]. Organisms that adjust their physiology in response to increasing cold (cold acclimation) can maintain function at low temperatures [4]. Therefore, the ability to cold-acclimate has a key role in shaping species distributions, particularly in determining altitudinal or latitudinal limits [5–7]. Strict thermal niches may restrict gene flow among populations adapted to different temperature regimes [8,9]. Consequently, adaptations that protect against temperature extremes may influence patterns of biodiversity and have important evolutionary implications in light of global climate change.

The ability to cold acclimate in insects correlates well with latitudinal distributions, with some high-latitude species exhibiting a greater capacity to acclimate [6,7,10–13]. The ability to cold acclimate is particularly advantageous to species experiencing strong seasonal temperature variation and those which need to overwinter in northern latitudes [14]. Much is known about the physiology and sensory cues involved with successful overwintering. However, our understanding of the genetic basis of cold tolerance is relatively poor. Few genes involved in the perception of cues for seasonal changes, the timing of mechanisms involved and the physiological changes associated with temperature challenges have been identified [15]. *Drosophila montana* is an ideal species for the study of the genetic basis of cold tolerance. This species belongs to the *virilis* group of *Drosophila*, and has a northern circumpolar high latitude distribution. It can survive at high altitude and successfully overwinters as an adult in northern Finland using strategies including reproductive diapause and cold acclimation, i.e. it is frigophilic [16].

A recent analysis of gene expression changes during cold acclimation in *D*. *virilis* and *D*. *montana* found that a number of differentially expressed genes were common to both species [17]. Although these species are relatively closely related, they have different cold tolerances as measured by chill coma recovery time [18]. This is likely to reflect thermal niche adaptation as *D*. *virilis* is typically found at lower latitudes (south from 35°N) than *D*. *montana* (30–70°N) [16]. Despite differences in baseline cold tolerance, both species are able to increase their cold tolerance after cold acclimation by a similar level [18].

Among the list of candidate genes obtained by Parker *et al*. [17] *myo-inositol-1-phosphate synthase (Inos)* stands out as a plausible candidate given what is known about its function. *Inos* encodes the enzyme *myo-inositol-1-phosphate synthase* which is the rate-limiting step in myo-inositol biosynthesis [19], the major metabolite produced during overwintering by *D*. *montana* [20]. Since *D*. *montana* is not a model species, studying the genetic basis of traits is relatively difficult as available genetic tools are limited. Here, we adopt an RNA interference (RNAi) approach to test the role of *Inos* in cold acclimation. By altering the expression of this gene, we successfully increased cold sensitivity in this normally cold hardy species and thus confirm its role in cold tolerance.

## Material and Methods

### Fly rearing

42 isofemale lines from Oulanka, Finland were established by Veltsos et al. [21]. Individuals from all these lines were isolated and intercrossed to produce a line with greater genetic variation in order to avoid potential issues of dealing with inbred lines such as differential susceptibility to RNAi. Lines were collected in 2009 and subsequently maintained at 19°C and constant light. Approximately 5 pairs from each line were collected and mated at random to form 20 new lines. Pairs from the F1 were then mixed to produce genetically diverse lines (essentially producing one mass bred line) for experimentation. Experimental stock flies were then reared in standard malt medium at 19°C and maintained under a 22:2 Light: Dark (LD) light cycle. Only female flies were used in cold-tolerance trials and for micro-injection. Females were collected under light CO_2_ anaesthesia within 24 hours of emergence to ensure virginity and kept in vials containing 20–25 flies for 14 days prior to experimental procedures to become sexually mature. Note the methodology decribed above is similar to that used by Parker *et al*. [17] to allow our results to be easily compared.

### Synthesis of double-stranded RNA

For both the target gene *Inos* and the control gene *LacZ*, (see below), fragments of approximately 800 bp in length were produced using a standard PCR protocol. Primers were designed to amplify regions avoiding intron/exon boundaries. Fragments were subsequently cloned into a pGEM-T Easy vector (Promega, Southampton, UK) according to the manufacturer’s instructions. This plasmid was then used as the template in a second round of PCR. The second set of primers contained a T7 promoter sequence at the 5’ end of both the forward and reverse primer. The resulting PCR products were approximately 500bp in length and contained the T7 promoter region to facilitate transcription of the double-stranded RNA (dsRNA). Synthesis of dsRNA, using T7 PCR products as a template, was carried out using the MEGAscript T7 Transcription Kit (Life Technologies Ltd., Paisley, UK) according to the manufacturer’s instructions. Double-stranded RNA was purified using the MEGAClear Kit (Life Technologies Ltd., Paisley, UK), eluted in a low-salt buffer, and quantified using a Nanodrop Spectrophotometer (Thermo Fisher Scientific, Loughborough, UK). We produced dsRNA for *Inos* and also the bacterial gene *lacZ* which was used as a control. The set of primers used for the first and second rounds of PCR are shown in S1 Table.

### Microinjection procedure

Prior to micro-injection, flies were anaesthetised under light CO_2_. For each target gene, three experimental blocks of micro-injection were carried out with approximately 200 flies injected per block. In each block, 100 flies were injected in the thorax with a total of 207 nl of dsRNA (4 μg/μl), of the target gene. The remaining 100 flies were injected with *lacZ* dsRNA. Microinjection was performed using a Drummond Nanoject II microinjector (Drummond Scientific Company, Broomall, USA). After injection, individuals were separated into small glass vials containing malt food and transferred to the appropriate incubator to assess their capacity to cold acclimate (see below).

### Cold acclimation trials

Injected flies were divided into two groups, each containing approximately 70 target and 70 control flies. One group was maintained at the control temperature of 19°C and the second at 5°C (22:2 L:D) for cold treatment. After 3 days all flies were transferred to fresh vials containing agar (10%) for moisture and exposed to a cold shock: -7°C for 16 hours in constant light. Flies were then transferred immediately to individual plastic containers for observation. Chill-coma recover time (CCRT) was recorded as a measure of cold tolerance (see Vesala *et al*. 2012a). A fly was considered to have “recovered” once standing on all six legs. This experiment was scored blindly to minimise observer bias. Mortality rate after the 3 days of acclimation before the cold shock was also recorded. A total of 385 flies were injected for the experiment divided equally in four groups (see below).

### Expression analyses

Real-time PCR was performed to confirm that dsRNA injections produced a change in the expression of the target gene. Expression analyses were performed only on flies maintained at 19°C due to high mortality in the 5°C treatment groups (see results). Flies were maintained at 19°C for two weeks as per the standard fly rearing protocol. Approximately 40 females were then injected with target dsRNA and another 40 with *lacZ* dsRNA. These females were transferred to new vials containing malt food and incubated at 19°C for 24 hours. Total RNA was extracted from 3 pools of 10 females per injection group (target and control) for each of 3 experimental blocks. RNA extraction was performed using the TRIzol Plus RNA Purification Kit (Life Technologies Ltd., Paisley, UK) and cDNA synthesized using TaqMan Reverse Transcription Reagents (Life Technologies Ltd., Paisley, UK) according to the manufacturer’s instructions

Quantitative real-time PCR was performed with a ABI Prism 7000 Sequence Detection System (Applied Biosystems) using Maxima SYBR Green/Fluorescein Master Mix (Life Technologies) according to the manufacturer’s instructions. The fluorescein acted as the passive reference dye, normalising the SYBR green signal between wells. Reactions were carried out in a final volume of 20 μl with oligonucleotides at a final concentration of 0.6 μM and 1 μl of cDNA template. We used the ΔΔCt method to convert raw expression data to normalised relative expression values, using the control (*LacZ* injected flies) treatment as the comparison group [22] and RP49 as the reference gene. Log_2_-transformed relative expression values were analysed using ANOVA in the statistical package R.

### Statistical analysis

All statistical analyses were performed with the statistical package R [23]. Data collected from the 3 separate trials in the cold acclimation experiments were analysed using generalised linear mixed models in the package lme4 [24]. The full model fitted temperature, injection and a temperature by injection interaction term as fixed effects, and experimental batch and “observer” were fitted as random effects. Both had significant effects on CCRT (p<0.001), and were therefore included in all statistical models. The statistical significance of random effects was determined by comparing the log-likelihood of the full model to one in which a random effect was omitted using a log-likelihood ratio test. The statistical significance of fixed effects was determined using Wald chi-square tests. If the interaction term was found to be non-significant (p>0.05), a reduced model without the interaction was used to determine significance of the other terms in the model. Note both full and reduced models are reported in S2 and S3 Tables. Mortality rate after the acclimation trials were compared pairwise using a Fisher’s exact test.

### Data Archive

All data obtained are presented in S4 Table.

## Results

### Cold acclimation phenotype

**Fig 1:**
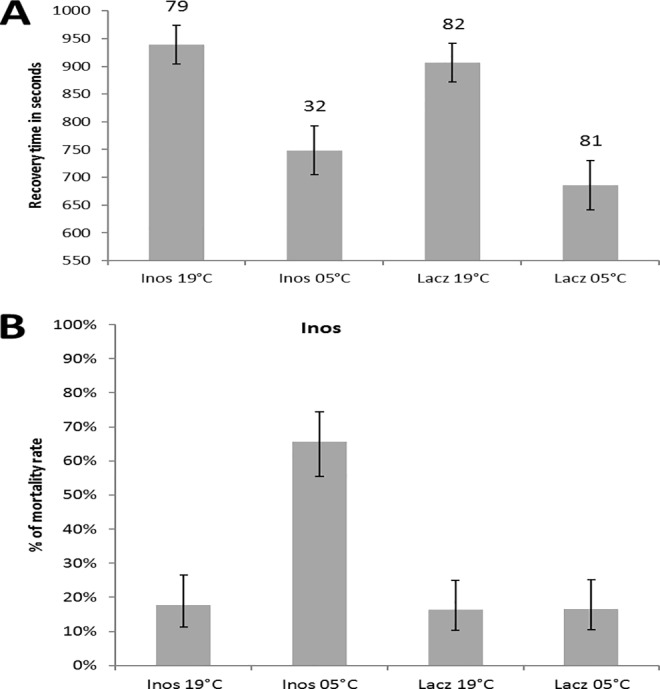
(A) Mean recovery time of females injected with either dsRNA*Inos* (target group) or dsRNA*lacZ* (control group) after 3 days of acclimation to either 19°C or 5°C followed by exposure to a cold shock. Numbers above bars represent sample size for each group and error bars represent the standard error. (B) Mortality rates of females injected with either dsRNA*Inos* (target group) or dsRNA*lacZ* (control group) after 3 days of cold acclimation at either 19°C or 5°C. The error bars represent the 95% binomial confidence interval.

Flies injected with dsRNA showed strong evidence of cold acclimation, with shorter CCRT after acclimation at 5°C (p<0.001 (Fig 1A, S3 Table) similar to what is observed in wild type flies [18]. Injection of dsRNA*Inos* however did not significantly affect CCRT (p = 0.258, Fig 1A, S3 Table). The interaction between temperature and injection was also non-significant (p = 0.755, S2 Table). However, flies injected with dsRNA*Inos* displayed a substantial increase in mortality rate (66%) when acclimated at 5°C (INOS-05°C: Fig 1B). The difference in mortality was significant in all pair-wise comparisons to the other 3 *Inos* groups (p<0.001 in all cases). However, 19°C dsRNA*Inos* injected flies (INOS-19°C) did not show any difference in mortality to the LACZ control groups (p = 0.387 to the LACZ-19°C and p = 0.379 to the LACZ-05°C). Such a high mortality rate in the INOS-05°C, but not in the INOS-19°C, group points to an important effect of *Inos* expression in altering cold tolerance.

### Gene expression

**Fig 2:**
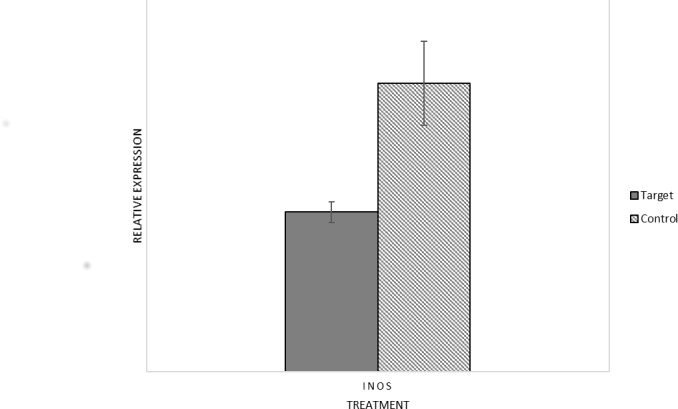
Expression of *Inos* relative to the expression of *RP49* in flies injected with the target dsRNA (solid grey bars) and flies injected with the control dsRNA (dashed bars). Error bars represent the standard error.

*Inos* expression was reduced following injection of dsRNA*Inos* when examined 24 hours after injection. The reduction was approximately 40% compared to control flies injected with dsRNA*lacZ* (p = 0.001, Fig 2).

## Discussion

Transcriptomics has provided a powerful method to identify candidate genes underlying the evolution and function of traits in non-model species lacking advanced genetic tools [25]. However, following up on transcriptomics can be challenging. Many variables can produce changes in gene expression so it is important to experimentally validate a role of potential candidate genes. Parker *et al*. [17] used an RNA-seq approach to identify genes which change expression during cold acclimation in *D*. *montana*, an extremely cold-adapted species. The ability to cold acclimate has clear fitness consequences and local adaptation to differing thermal regimes is critically important to understanding climate change and species distribution and abundance [9].

By using an RNAi injection technique we were able to examine the effect *Inos* has on the ability of flies to cope with a cold shock with or without a period of acclimation. Our prediction was that injection of dsRNA complementary to *Inos* would lead to a reduced ability of flies to acclimate leading to a reduced ability to cope with cold shock.

Even though injection of dsRNA*Inos* did not alter CCRT, either overall or in interaction with the cold treatment, our cold acclimation response results should be considered alongside our finding that flies treated with injection of dsRNA*Inos* showed a large increase in mortality during the cold acclimation treatment. Two thirds of the flies treated with dsRNA*Inos* died during the treatment. This reduced the sample size for these groups, and it is perhaps likely that the surviving flies represent a biased subset of flies less susceptible to RNAi treatment [26] or were otherwise more cold-tolerant.

Our qPCR results showed that injection of dsRNA*Inos* produced a knock-down of *Inos* expression as expected, reducing gene expression by approximately 40%. The expression levels were measured here only in flies at 19°C as the high mortality rate of flies acclimated at 5°C prevented us from quantifying gene expression in that condition.

Our finding that manipulation of *Inos* increased cold-induced mortality in this cold tolerant species strongly supports our hypothesis and the results of Parker *et al*. [17] that *Inos* is involved in increasing cold tolerance during cold acclimation. *Inos* encodes the enzyme *myo-inositol-1-phosphate synthase*, which is part of the inositol biosynthetic pathway, catalysing the conversion of D-glucose-6-phosphate into L-myo-inositol-1-phosphate, the first committed step of *de novo* inositol synthesis [19]. Inositol compounds are important precursors for structural lipids (phosphatidylinositols) which are important components of eukaryote cell membranes [27,28]. Changes to cell membrane composition are critical for adaptation to temperature as they allow cells to maintain their osmotic balance and function [15,29,30]. We suggest that by increasing expression of *Inos D*. *montana* increases the amount of myo-inositol, changing the composition of their cell membrane, which results in an increase in cold tolerance.

In our study we were able to successfully use dsRNA injections to alter gene expression in *D*. *montana*, even though this technique has had a very limited effect in *D*. *melanogaster* [31,32]. Recently, Scott et al. [33] reviewed the effectiveness of dsRNA injections across several insect groups and found that it varies greatly among taxa, with *D*. *melanogaster* representing the extreme end of poor performance while another dipteran *Aedes aegypti*, performs much more successfully. The reasons for this variation are unknown but may be related to rapid evolution of components of the RNAi anti-viral response amongst species [34]. Our study shows that variation in effectiveness of introducing dsRNA by injection can vary within a single genus. This is an important finding as many other species of *Drosophila* have now been sequenced, but lack developed functional genetic tools. Finding that dsRNA injections are effective in *D*. *montana* opens the door for this relatively simple and inexpensive way of manipulating gene expression in other non-model *Drosophila* species.

Overall, our study demonstrates that *Inos* is important for cold tolerance in *D*. *montana*. Further studies are necessary to fully understand the molecular mechanism by which *Inos* affects cold tolerance. For instance, using the CRISPR/CAS9 system [35,36] to produce *D*. *montana* transgenic lines should allow for more precise manipulation of gene expression that could provide these answers.

*Inos* has not been previously implicated in increasing cold tolerance in non *D*. *virilis* group species. One implication from this is that the involvement of *Inos* in cold tolerance is specific to the *virilis* group flies. This is perhaps unlikely because *Inos’* final product, myo-inositol, has been shown to accumulate in response to the onset of winter in several other insect species [37,38], including other dipterans [39]. Taken together these finding suggest that *Inos* may influence cold tolerance in a wide range of species, but more extensive comparative studies are needed to explore this further.

## Supporting Information

S1 Table Sequence of the primers used for the molecular experiments. (DOCX)

S2 Table Significance of acclimation temperature (19°C or 5°C), injection (dsRNA*Inos* or dsRNA*lacZ*) and their interaction (full model) on chill-coma recovery time. Note experiment batch and recorder were fitted as random effects. Significant values are presented in bold.
(DOCX)

S3 Table Significance of acclimation temperature (19°C or 5°C), injection (dsRNA*Inos* or dsRNA*lacZ*) (reduced model) on chill-coma recovery time. Note experiment batch and recorder were fitted as random effects. Significant values are presented in bold.
(DOCX)

S4 Table Table containing the raw data obtained in the present study. (XLSX)
