# Supplementary material for: Inducing Cold-Sensitivity in the Frigophilic Fly Drosophila montana by RNAi
Source: PLoS One. 2016 Nov 10;11(11):e0165724. doi: 10.1371/journal.pone.0165724 (PMC5104470; doi:10.1371/journal.pone.0165724)
Supplement: S1 Table — (DOCX) [file pone.0165724.s001.docx]

|  | **Primer name** | **Sequence 5' to 3'** |
| --- | --- | --- |
| **RNA interference** | | |
| *lacZ* | lacZ_T7_F | TAATACGACTCACTATAGGGAGAATCCGACGGGTTGTTACT |
|  | lacZ_T7_R | TAATACGACTCACTATAGGGCACCACGCTCATCGATAATTT |
| *Inos* | INOS_MONT_4_F | GTGCAACCGGGTCTGAATAC |
|  | INOS_MONT_4_R | TTGCTCCAAAGACAGATGCG |
|  | INOS_T7_F2 | TAATACGACTCACTATAGGGTAAGCAGTTCCGCTCCAAGG |
|  | INOS_T7_R2 | TAATACGACTCACTATAGGGTGCTCCAAAGACAGATGCGA |
| **Real-Time PCR** | | |
| *Inos* | qINOS_F3 | CCCTTGGGTGCCCTTTA |
|  | qINOS_R3 | AGCACGCTGGCTGAAGAG |
| *RP49* | qRp49_F1 | CCAGCATACAGGCCCAAGAT |
|  | qRp49_R1 | GCATATCGATCCGACTGGTG |
