# Supplementary material for: Inducing Cold-Sensitivity in the Frigophilic Fly Drosophila montana by RNAi
Source: PLoS One. 2016 Nov 10;11(11):e0165724. doi: 10.1371/journal.pone.0165724 (PMC5104470; doi:10.1371/journal.pone.0165724)
Supplement: S3 Table — Note experiment batch and recorder were fitted as random effects. Significant values are presented in bold. (DOCX) [file pone.0165724.s003.docx]

|  | Wald chi-square | Df | p-value |
| --- | --- | --- | --- |
| Temperature | 33.0469 | 1 | **9.00 x 10^-9^** |
| Injection | 1.2813 | 1 | 0.258 |
